# Online Information of COVID-19: Visibility and Characterization of Highest Positioned Websites by Google between March and April 2020—A Cross-Country Analysis

**DOI:** 10.3390/ijerph19031491

**Published:** 2022-01-28

**Authors:** Anna Kłak, Jolanta Grygielska, Małgorzata Mańczak, Ewelina Ejchman-Pac, Jakub Owoc, Urszula Religioni, Robert Olszewski

**Affiliations:** 1 Department of Environmental Hazards Prevention, Allergology and Immunology, Medical University of Warsaw, st. Banacha 1a, 02-091 Warsaw, Poland; 2 Gerontology, Public Health and Didactics Department, National Geriatrics, Rheumatology and Rehabilitation Institute, st. Spartańska 1, 02-637 Warsaw, Poland; jgrygielska@wp.pl (J.G.); malgorzata.manczak@spartanska.pl (M.M.); ewelinae@o2.pl (E.E.-P.); jakub.owoc@spartanska.pl (J.O.); robert.olszewski@spartanska.pl (R.O.); 3 Collegium of Business Administration, Warsaw School of Economics, st. Madalińskiego 6/8, 02-513 Warsaw, Poland; urszula.religioni@gmail.com; 4 School of Public Health, Centre of Postgraduate Medical Education of Warsaw, st. Kleczewska 61/63, 01-826 Warsaw, Poland; 5 Department of Ultrasound, Institute of the Fundamental Technological Research of the Polish Academy of Sciences, st. Pawinskiego 5B, 02-106 Warsaw, Poland

**Keywords:** COVID-19, Coronavirus, SARS-CoV-2, fake news, misinformation, Internet, Google, online information, infodemic, infodemiology

## Abstract

Background: The WHO has used the term “infodemic” to describe the vast amount of false and true information that was making it difficult for people to find reliable information when they needed it. The infodemic spreads faster than COVID-19 itself. The main objective of the study was to characterize and analyze content about COVID-19 returned by Google during the pandemic and compare it between countries. Methods: The study was conducted between 30 March and 27 April 2020. The information was searched through local Google websites using the “COVID-19”, “Coronavirus”, “SARS-CoV-2” and “fake news” keywords. The search was conducted in Australia, France, Germany, Italy, Poland, Singapore, Spain, UK and the USA. The total number of the analyzed webpages was 685. Results: The most frequent types were News websites 47% (324/685) and Governmental 19% (131/685) while the least were Health portals 2% (17/685) and Scientific journals 5% (35/635), *p* < 0.001. United States and Australia had the highest share of Governmental websites. There was a positive correlation between the amount of preventive information and a number of SARS-CoV-2 infections in countries. The higher the number of tests performed, the higher was the amount of information about prevention available online. Conclusions: Online information is usually available on news and government websites and refers to prevention. There were differences between countries in types of information available online. The highest positioned (the first 20) websites for COVID-19, Coronavirus and SARS-CoV-2 keywords returned by Google include true information.

## 1. Introduction

Globalization, common access to the Internet and the rapid development of social media make fake information easy to spread. The phenomenon is so widespread that the English phrase “fake news” has made it its way to other languages. “Fake news” is defined as: “fabricated information that imitates news media content in form but not in organizational process or intent, which overlaps with other information disorders, such as misinformation (false or misleading information) and disinformation (false information deliberately disseminated to deceive people)” [1]. False information is a threat that impacts reality. Thus, it has also attracted the attention of researchers who for years now have been analyzing the role that fake news plays, how it spreads and ways to eliminate it [2,3,4,5,6].

Fake news related to health has been spreading for quite some time. Most of it refers to anti-vaccination movements [7,8], teeth strengthening and whitening at home [9], oncology treatments [10]. Fake news may provide patients and their families with false hope and reason for questioning existing medical knowledge or for exerting pressure on physicians [10].

False information may be particularly harmful during pandemics such as in the SARS-CoV-2 pandemic that has infected millions of people and is still spreading [11]. Searching for information during a pandemic about prevention, treatment and recovery is a natural reaction. What makes people fearful is the COVID-19 mortality rate. The death toll (27 April 2020) is hundreds of thousands around the world at approximately 7% of all infections, and a Case Fatality Ratio (CFR) of 18.8% [12,13].

In the situation of a rapidly developing pandemic and the lack of a vaccine or medication against COVID-19, news about prevention quickly makes it to the social media such as Facebook, Twitter, Whatsapp, Tik Tok or Instagram. Some of the information is false and may be even harmful [6,14]. Therefore, the amount of fake news appearing during the study period was limited by the actions of official entities (state and regional authorities, public health institutes, scientific societies, World Health Organization (WHO), etc.) providing verified information. At the same time, these institutions provided information on how to check information about a pandemic emerging in the public space. Among the most frequently appearing fake news was information on various methods of preventing infection, such as consuming large amounts of vitamin D, vitamin C, garlic, drinking water every 15 min [15,16]. Conspiracy theories also appeared, among them, the theory about the responsibility of 5G technology for the spread of the virus, which contributed to the destruction of the technology’s masts, and the theory that vaccination against COVID-19 serves to subjugate citizens [15,16].

The first official cases of pneumonia of unknown etiology in the city of Wuhan were reported by Chinese authorities to the WHO on 31 December 2019. A new type of virus responsible for pneumonia was identified in January 2020 and classified as 2019-nCoV. Just several weeks later, similar cases had been reported in Thailand, Japan and South Korea [17]. The first European cases were in Italy on 31 January 2020. The WHO Information Network for Epidemics (EPI-WIN) for providing reliable information began operating on the same day [18]. On 11 February, the WHO also introduced a new classification, renaming 2019-nCOV to SARS-CoV-2 and naming the disease caused by the virus COVID-19 [19]. New ICD-10 and ICD-11 codes were implemented and COVID-19 became an official cause of death [20,21].

The WHO classified COVID-19 as a pandemic on 11 March 2020 [22]. A month earlier, the WHO had used the term “infodemic” in one of its daily reports to describe the vast amount of both false and true information that was making it difficult for people to find reliable information when they needed it. The infodemic was spreading faster than COVID-19 itself [23,24]. Infodemiology is a science around the distribution and determinants of information in electronic media. Infodemiological data are usually collected and analyzed in almost real time. Some examples of infodemiological data applications are in analyzing search engine queries to predict disease outbreaks (e.g., influenza) and identifying and monitoring public health online publications, e.g., anti-vaccine websites. Infodemiology can provide valuable information on population health behaviors [25,26].

In this study, researchers investigated online information related to the SARS-CoV-2 coronavirus and the COVID-19 diseases as returned by a Google keyword search. They chose countries on four continents: Europe (Poland, UK, Spain, Italy, France and Germany), North America (USA), Asia (Singapore) and Australia. There was an increase in COVID-19 cases in these countries during the April 2020 period. These countries also differed in the percentage of deaths among those infected. Compared to the incidence on 1 April 2020, at the end of the month in countries such as Poland and the UK, the increase in infections was 5–6 times. In Italy, Spain, France and Germany, the increase was more than double. The largest differences were observed in Singapore (more than a 14-fold increase), but a significant increase in the incidence did not occur until 20 April. The increase in infections was the slowest in Australia (133%); usually it was a double-digit daily increase, with the highest recorded amounting to 266 new cases (2.04). The number of deaths varied from country to country. The highest percentage of deaths as of 1 April 2020 in relation to cases was recorded in Italy (11.9%), Spain (9.0%), Great Britain (8.7%), France (7.1%). These values were higher than the world average (5.2%). In other countries, this ratio was lower than the world average and amounted to: USA: 2.9%, Poland: 1.8%, Australia: 0.5% and Singapore: 0.3% [11,13].

The aim of the study was to characterize and analyze the pages of the first 30 search engine results (SERP) for each country publicly available during the pandemic, and to compare them between countries. Detailed objectives included:

- Characterization and analysis of websites typology by Google based on “COVID-19”, “Coronavirus”, “SARS-CoV-2” and “fake news” key words;
- Characterization and analysis of online information returned by Google based on “COVID-19”, “Coronavirus” and “SARS-CoV-2” key words;
- Calculating frequency of fake news on “COVID-19”, “Coronavirus”, “SARS-CoV-2” key words;
- Characterization and analysis of online information returned by Google based on “fake news” key words;
- Analysis of associations between results and epidemiological data on COVID-19, such as: number of deaths, number of infections, number of SARS-CoV-2 tests performed;
- Characterization and analysis of online information returned by Google based on “COVID-19”, “Coronavirus”, “SARS-CoV-2” and “fake news” about celebrities, religion and testimonials;
- Analysis of the *Journal of the American Medical Association* (JAMA score).

The research question was whether there were differences between the countries in content available online about COVID-19.

## 2. Materials and Methods

### 2.1. Websites

The study was conducted in 2020 between March 30 and April 27 using the methodology employed by Arif N. et al. [7] for analyzing content about immunization. Authors used “vaccines” and “autism” keywords in Google between June and September 2017 and categorized the websites. The typology included: Commercial (C), Government (G), Health portal (HP), News (N), Non-profit (NP), Professional (P), Scientific journals (SJ) and “others” (O).The JAMA score was used (for the presence of the following information: author, date, references, owner of website) and the webpages were annotated according to the following features: (1) the name of the vaccine mentioned; (2) the overall stance on vaccines (positive, negative, or neutral); (3) the chemicals or adjuvants mentioned; (4) whether the page mentioned complementary and alternative medicine (CAM); (5) whether religion was mentioned; (6) whether the page contained a testimonial; (7) whether a celebrity was mentioned [7].

This study was carried out according to SRQR guidelines (standards for reporting qualitative research) [27].

The research was carried out using both Google Chrome and Firefox browsers without any add-ons. All cookies and the browser history were deleted prior to the search process to avoid any bias in the results [7]. The researchers were aware that Google may identify the user’s location using their IP address and so influence the results, so they did not use a VPN while searching. The following searches were conducted: Poland (google.pl), France (google.fr), Italy (google.it), USA (google.com), UK (google.co.uk), Singapore (google.com.sg), Germany (google.de), Spain (google.es), Australia (google.com.au). All SERPs for each country and one keyword were analyzed on the same day. This part of the methodology is important in order to calculate the return frequency of websites in relation to their type. If the content returned was too extensive to analyze in one day (one keyword for a country), the links to the first 30 SERPs were copied to Excel and analyzed over two days.

As the search process was performed in Poland, the research team deleted the browser history and all cookies, and reset Google to the selected country and language. Although the keywords were typed in English, Google was set to return results in the national languages.

### 2.2. Keywords

The country Google search engines were used and four key words: “COVID-19”, “Coronavirus”, “SARS-CoV-2” and “fake news”. All but one national language in the study use the same spelling for “coronavirus”. The local word “koronawirus” was used for Poland. In Singapore, which has four official languages including English, the returned results using “coronavirus” were considered equal and comparable to other countries. It was decided to use only those keywords as we planned to obtain a sample of the websites returned independently for each expression. Thus, we did not to use questions such as “mitigations measures?”, “auto tests?”, “how to treat COVID-19” or “how to protect against coronavirus” because the results would be different depending on an exact question. We decided to use the search terms “COVID-19”, “Coronavirus”, “SARS-CoV-2” and “fake news” as this best represents what the lay public would search on the Internet. The researchers had basic and advanced language skills in the languages of the analyzed countries. Unclear content was translated into English or was consulted upon with translators.

The researchers deliberately used the keyword “fake news” because at the time the study was conducted, it was popular on the web to comments on fake news about the COVID-19 pandemic. However, a detailed analysis showed that some of the fake news about the COVID-19 pandemic was not debunked, but accepted as real information.

### 2.3. Content Analysis

Three researchers analyzed the first 30 SERPs for three different countries at the same time. There was no more information on the Internet about the topic at the time the study was carried out. By design, 30 SERPs were taken into account after the initial research. After compiling items for different countries in their national languages (in Singapore-English), it turned out that getting 30 addresses for each keyword is impossible. In the end, the first 20 SERPs were taken into account, which for some countries and for this reduced number was impossible to obtain (fake news: Spain-16 pages, Poland-13, see: Table 1). Each researcher had to analyze 4 key words for each country, i.e., 120 SERPs and 360 SERPs in total. Each researcher analyzed one keyword a day for a country. Whenever the content was too extensive, the links to SERPs were copied to Excel and investigated the next day. The overall number of the analyzed SERPs reached 685 with the following numbers for each of the countries: Poland: 73, France: 80, Italy: 80, UK: 61, Singapore: 81, Germany: 80, Spain: 76, Australia: 80, USA: 74.

Using a 0–1 system, the researchers assessed whether each type of information appeared on the SERP, where 0 means no information, and 1 means it was present. Moreover, each SERP was rated according to webpage typology. The fact that the information on the website was “fake news” was also assessed using 0–1, where 0 means real information and 1 means fake news. The researchers, by consensus, judged whether the information was fake news or not, on the basis of information posted on websites that were generally considered reliable. Such websites include: the World Health Organization, WHO; the Centers for Disease Control and Prevention, CDC; the National Health Service, NHS; or national health authorities. Additionally, it was necessary for the team to agree on the comparability of the published information in different countries on the same topic. A structured study protocol (flowchart) is presented in Table 1 and the Supplementary Materials (Tables S1 and S2).

Researchers have jointly defined the scope of the analysis based on the information on COVID-19 available in the public domain of the Internet methods used by Arif N. et al. [7]. Each website was assessed according to the information it contained. The following content was analyzed: information on quarantines, disease/infection symptoms, disease/infection risk factors, disease/infection consequences; virus transmission routes; virus incubation period; virus carrier; disease treatment; prevention measures prior to infection; alternative/supplementary medicine–unconventional or “home” treatment (including attitudes towards alternative medicine: positive, negative, neutral); epidemiological data (number of cases, deaths etc.); whether the page contained a testimonial (e.g., a personal story); whether a celebrity was mentioned; whether a religion was mentioned; others such as: regulations, services, economy, information on online fake news. The “other” category also included information on disease etiology. The etiology was not initially considered a separate category; however, numerous websites included speculation on how the pandemic started. All such speculation was eventually classified as “other”. All the websites returned by Google using the “COVID-19”, “Coronavirus” and “SARS-CoV-2” keywords were judged as true or false according to the scientific knowledge provided by organizations such as the CDC; NHS; Chief Sanitary Inspectorate “Główny Inspektorat Sanitarny, GIS”; National Health Fund “Narodowy Fundusz Zdrowia, NFZ”; Ministry of Health “Ministerstwo Zdrowia, MZ”; WHO, etc. The results for “fake news” were checked for whether the false information on the website had been deemed false. Fake news was defined as information inconsistent with the knowledge and information provided by the WHO, CDC, NHS or national health authorities. The SERPs for the “COVID-19”, “Coronavirus” and “SARS-CoV-2” keywords did not contain any websites on alternative medicine; thus, it was not included in further analysis.

### 2.4. Websites Typology

Each website was classified according to the typology used in other studies [7]:

- Government (G): websites of governmental bodies, local authorities; e.g., www.whitehouse.gov, www.epa.gov; (accessed on 30 March 2020)
- Health Portal (HP): websites with information on a variety of health topics, e.g., www.medscape.com (accessed on 30 March 2020);
- News (N): websites of newspapers, magazines or TV; e.g., www.cbsnews.com (accessed on 30 March 2020), www.nytimes.com (accessed on 30 March 2020);
- Non-Profit (NP): websites of non-profit organization, e.g., https://choice.npr.org (accessed on 30 March 2020);
- Professional (P): websites created by health professional organizations (medical school, clinic/hospitals, medical boards); e.g., https://sph.nus.edu.sg (accessed on 30 March 2020);
- Commercial (C): websites selling drugs, supplements or other; e.g., https://www.diagnostictechnology.com.au/ (accessed on 30 March 2020);
- Scientific journal (SJ): websites of academic journals, e.g., www.thelancet.com (accessed on 30 March 2020).

An exception was made for websites such as: https://www.cdc.gov (accessed on 30 March 2020), https://www.nih.gov (accessed on 30 March 2020), https://www.pzh.gov.pl (accessed on 30 March 2020), https://www.aifa.gov.it/ (accessed on 30 March 2020) that used a governmental domain but were classified as Professional. The first two News (N) websites were analyzed for each of the keywords.

### 2.5. Selection of Countries

The countries were from four continents: Europe (Poland, UK, Spain, Italy, France and Germany), North America (USA), Asia (Singapore) and Australia. Out of these nine countries, six had the highest number of infections as of 27 April 2020: USA, Spain, Italy, France, Germany and UK [11]. It was initially intended to include four countries but eventually extended to include USA, Singapore and Australia and make it more representative and comparable across continents. The European countries had the highest number of COVID-19 cases. Poland was added as the authors’ home country.

### 2.6. Inclusion and Exclusion Criteria

The researchers originally assumed to analyze the first 20 websites returned on each search engine result page (SERP). However, in some countries, keywords such as “fake news” returned only a few SERPs concerning the pandemic. Links to “Wikipedia”, “top stories”, “ads”, the WHO site, paid content and sites requiring registration were excluded. The links on the SERPs that referred to the keywords were copied to a standard Excel spreadsheet and described in detail. The SERPs returned by Google from the “fake news” keyword were included only if they referred to the COVID–19 pandemic.

### 2.7. JAMA Score

The JAMA score was based on information such as author (authorship), date (currency), financial ownership (disclosure) and references (attribution) [7,28]. Researchers evaluated each of these four aspects and either awarded a point or not. The JAMA score is the sum of the points awarded to a given website (for information relating to each of the four categories). The evaluated website could therefore receive between 0 and 4 points. In the JAMA evaluation, 1 point is insufficient information, 2–3 points are partially sufficient information and 4 points represent completely sufficient information [7,28].

### 2.8. Epidemiological Data and Statistical Analysis

Search results were tabulated with numbers and percentages. The COVID-19 epidemiological data for each analyzed country were read from the Worldometer website for the period from 1–27 April 2020 and presented as numbers [11]. The data covered: number of deaths, number of infections, number of SARS-CoV-2 tests performed. A Spearman rank correlation coefficient was used to determine associations between the search results and COVID-19 epidemiological data [29,30,31]. Correlation coefficients were calculated between COVID-19 epidemiological data and the frequency of coronavirus articles in the websites, and the frequency of information types in the websites. The correlation coefficient was considered statistically significant at *p* < 0.05. The JAMA score was used to assess the reliability of each website, where the score considers the author, date, financial ownership and references at 1 point for each. The range of JAMA score values is then from 0 to 4 [25]. The JAMA scores are presented as medians and interquartile ranges (IQR). Statistical analyses were performed with STATISTICA v 13.1 (Dell Inc., 2016, Tulsa, OK, USA).

## 3. Results

### 3.1. Characterization and Analysis of Websites Typology by Google Based on the Keywords “COVID-19”, “Coronavirus”, “SARS-CoV-2” and “Fake News”

The detailed analysis covered 685 websites in nine countries. The most frequent types were news (47%, *n* = 324) and governmental (19% *n* = 131) websites. Governmental pages accounted for the majority of the websites in the United States (35%, 26/74). Australia had roughly similar percentages of news 40% (32/80) and governmental 38.7% (31/80) websites. Commercial websites had substantial shares in Singapore 24.6% (20/81), United States 17.5% (13/74) and Poland 16.4% (12/73). Professional websites had the highest shares in Spain 14.4% (11/76), Germany 18.7% (15/80) and Italy 16.2% (13/80). Scientific journal websites had the largest share in Singapore and the United States (both with 14.8%). Health portals and non-profit websites rarely referred to the 4 keywords, at 2.4% (17/685) and 4.5% (34/685) respectively. Detailed information is presented in Table 1.

News websites were the most common type in six of the nine analyzed countries. In three, Poland, Germany and France, their share exceeded 50% (37/73; 51/80; 59/80). United States and Australia had the highest share of governmental websites. Detailed information is presented in Table 2.

**Figure 1:**
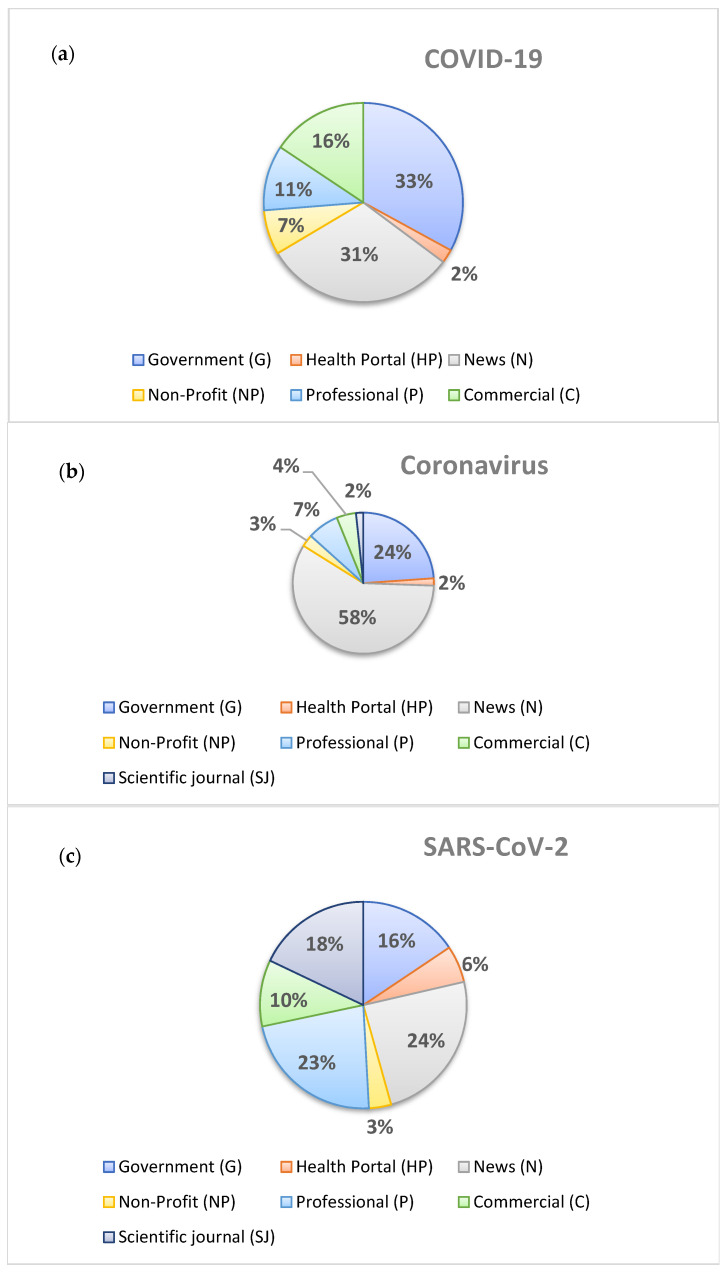
Frequency of key words on different types of webpages (all countries): (**a**) COVID-19; (**b**) Coronavirus; (**c**) SARS-CoV-2.

Out of the 685 SERPs for the “COVID-19”, “Coronavirus”, “SARS-CoV-2” and “fake news” keywords, 26.1% (179/685) referred to “COVID-19”, 26.2% (180/685) to “Coronavirus” and 25.2% (173/685) to “SARS-CoV-2”. Of the 179 SERPs for the “COVID-19” keyword, governmental websites accounted for 33% (59/179) and news websites for 31% (56/179). Of the 180 SERPs for the “Coronavirus” keyword, news websites accounted for 58% (105/180) and governmental websites for 24% (43/180). Of the 173 SERPs for the “SARS-CoV-2” keyword, news websites accounted for 24% (42/173), professional websites for 22% (38/173), scientific journal websites for 18% (31/173) and governmental websites for 15% (26/173). Detailed information is presented in Figure 1a–c.

### 3.2. Characterization and Analysis of Online Information Returned by Google Based on the “COVID-19”, “Coronavirus” and “SARS-CoV-2” Keywords

The analysis covered 532 websites that referred to the “COVID-19”, “Coronavirus”, “SARS-CoV-2” keywords. The sites included the following information: prevention against infection 32.7% (174/532), epidemiological data 31.3% (167/532), symptoms of diseases/infection 26.1% (139/532), diseases/infection risk factors 24% (128/532), transmission routes 23.6% (126/532). There was a large number of websites classified as other, 48% (256/532). They included information such as regulations during the pandemic, services or the economy.

**Figure 2:**
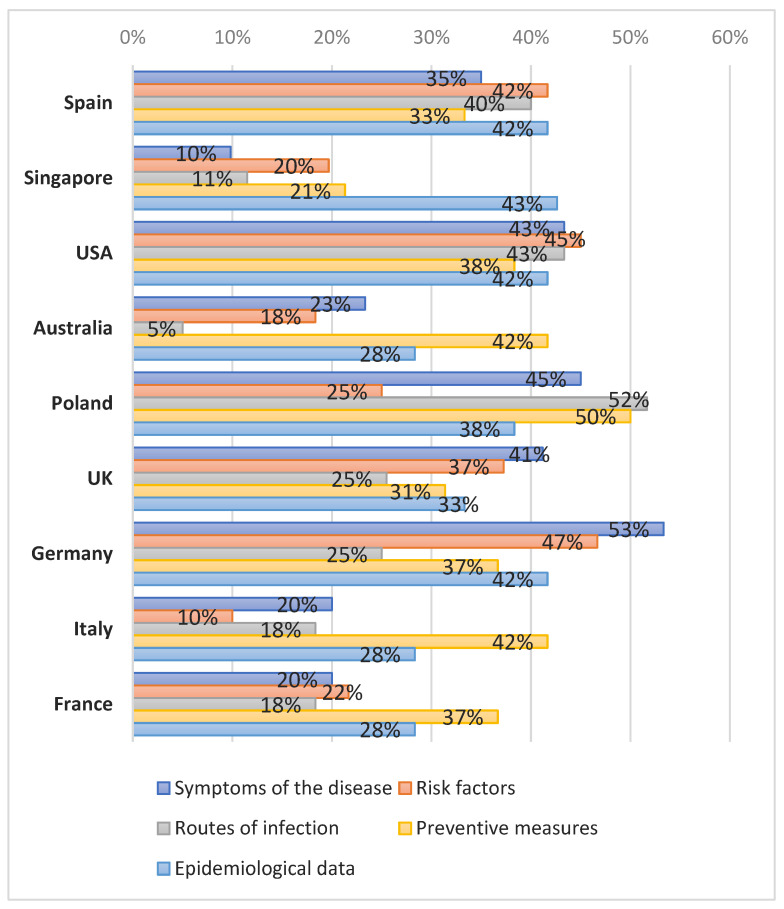
Frequency of information types in each country, *N* = 532.

The frequency of websites with information on epidemiology ranged from 28% (17/60) for Australia to 42% (25/60) for Germany. For the websites describing preventive measures, the percentage was between 21% (13/61) for Singapore and 50% (30/60) for Poland. Detailed results for each country are presented on Figure 2.

### 3.3. Calculating the Frequency of Fake News Referrals from the “COVID-19”, “Coronavirus” and “SARS-CoV-2” Keywords

Only 2.6% (14/532) of the websites referred to information on fake news. The research team judged 531 of the 532 websites reliable/correct (based on the scientific knowledge from GIS, NFZ, MZ, WHO, etc.). One website contained questionable information. Details are presented in Supplementary Materials (Table S1).

### 3.4. Characterization and Analysis of Online Information Returned by Google Based on the “Fake News” Keyword

Of the 153 SERPs that made references to “fake news”, 89.5% (137/153) contained information that denied the fake news circulating around the internet and social media. Three websites did not contain any denials, while one had information that had to do with Spanish politics and could not be precisely classified. Most of the 153 pages, 98% (150/153), referred to COVID-19, 34.6% (53/153) referred to preventive measures, and 36% (55/153) contained information that could only be classified as other (regulations during the pandemic, services, the economy, origins of the virus). Detailed information is presented in the Supplementary Files (Table S2).

**Figure 3:**
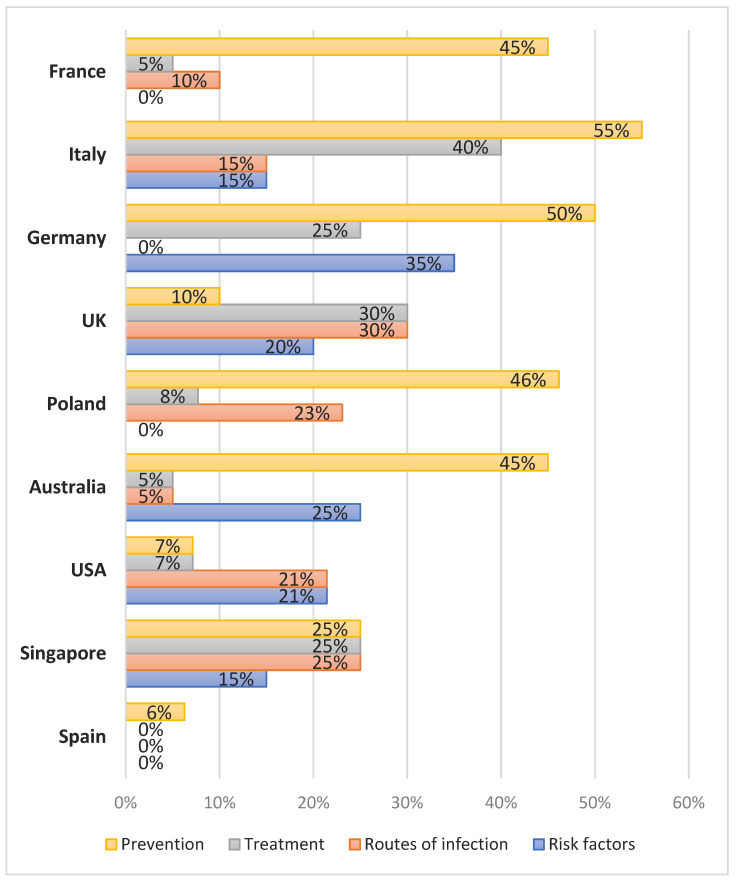
Most frequent types of information on fake news referring to COVID-19 according to countries.

In France, Italy, Germany, Poland and Australia, the information returned about fake news on the COVID-19 pandemic usually referred to prevention (45–55%), while in USA and Spain, the percentages were just 7% (1/14) and 6% (1/16), respectively. Details are presented in the Supplementary Files (Table S2) and in Figure 3.

Of the 153 SERPs for the “fake news” keyword, researchers identified 448 pieces of information that referred to fake news on the COVID-19 pandemic (debunked fake news) and grouped them into the 9 most frequent topics: coronavirus was created by peoples “miraculous” ways to prevent infection, routes of transmission, underestimating the virus, “harmful” technologies causing the pandemic, children, youths and black people being resilient to coronavirus infections, “effective” treatment methods, local sensations related to the virus, instructions on how to avoid fake news, and others. The most frequent topic was “miraculous” ways of preventing infection, 32% (145/448), followed by local sensations, 13% (56/448) and “effective” treatment methods, 10% (45/448). Detailed data are presented in Table 3 while Table 4 includes examples of debunked fake news.

### 3.5. Analysis of Associations between Results and Epidemiological Data on COVID-19

The research team attempted to find associations between the frequency of articles on coronavirus in the different types of websites and epidemiological data for countries, i.e., number of tests performed, number of infected people or number of deaths. The Spearman’s correlation coefficients were high (0.5–0.6); however, the low number of countries made the results statistically insignificant.

**Figure 4:**
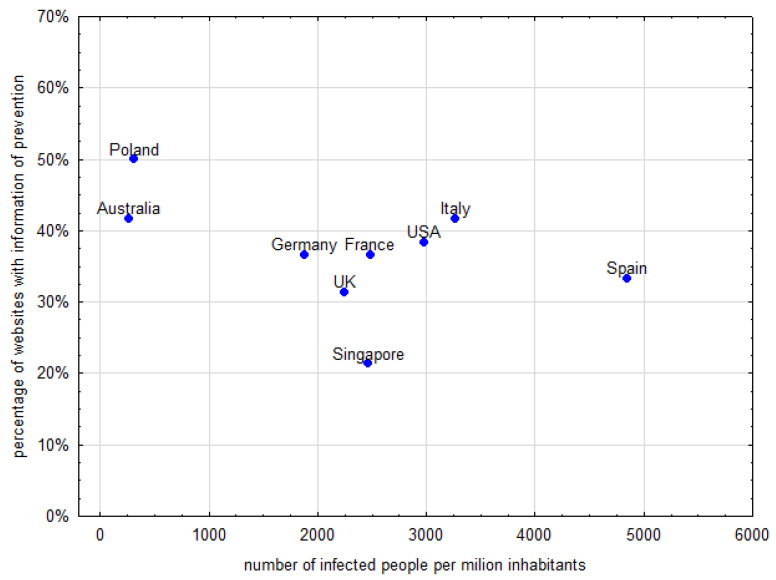
Change in a percentage of websites with information on prevention with number of infected people per million inhabitants.

Another analysis investigated the association between the frequency of information types and epidemiological data. The frequency of different content types did not correlate with data on the number of tests, number of infected people or deaths. There was an association between information on preventive measures and the number of people infected. Australia and Poland had low numbers of infections and had the largest amount of information about prevention. Detailed data are presented on Figure 4.

**Figure 5:**
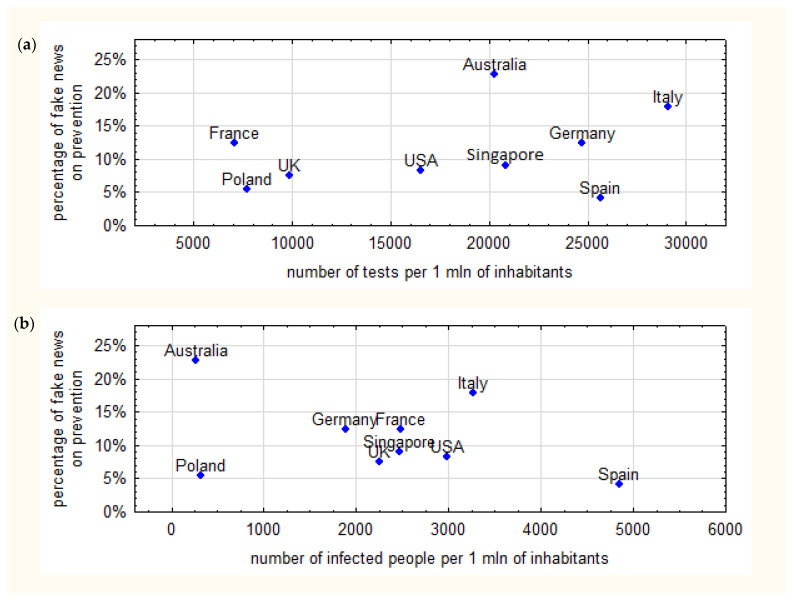
(**a**,**b**) Scatter plots of (**a**) number of tests and percentage of fake news on prevention and (**b**) number of infected people and percentage of fake news on prevention.

The information about fake news that referred to the COVID-19 pandemic was dominated by preventive measures. Researchers checked whether the frequency of fake news about prevention was correlated with epidemiological data and observed that the higher the number of tests, the higher the frequency of information on fake news referring to prevention. On the other hand, it was found that the higher the number of infections, the lower the amount of information on fake news referring to prevention. The scatter plots on Figure 5 present the detailed data.

### 3.6. Characterization and Analysis of Online Information Returned by Google Based on the “COVID-19”, “Coronavirus”, “SARS-CoV-2” and “Fake News” Keywords about Celebrities, Religion and Testimonials

Of the 532 SERPs for the “COVID-19”, “Coronavirus” and “SARS-CoV-2” keywords, only 3% (14/532) referred to information on testimonials or celebrities and less than 1% (2/532) to religion (Supplementary Files, Table S1). Of the 153 SERPs for the “fake news” keyword, 1% (18/153), 8% (13/153) and 2% (3/153) referred to testimonials, celebrities and religion, respectively (Supplementary Files, Table S2). Table 5 presents examples of information on celebrities, religion and testimonials.

### 3.7. JAMA Score

**Figure 6:**
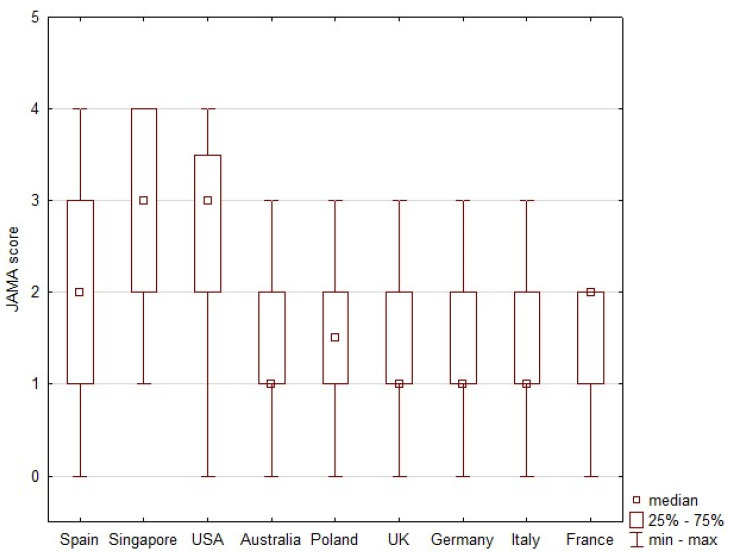
JAMA score of webpages on search engine result pages (SERPs).

The median JAMA score for all SERPs is shown in Figure 6. The Singapore and USA SERPs have a significantly higher JAMA score than any other SERPs. The scores for Australia, Poland and UK, Germany, Italy and France were comparable.

## 4. Discussion

### 4.1. Characterization and Analysis f Websites Typology by Google Based on the “COVID-19”, “Coronavirus”, “SARS-CoV-2” and “Fake News” Keywords

Daily messages that inform citizens about the current epidemiological situation and recommend self-isolation cause social concerns and anxiety. Meanwhile, the lack of reliable information encourages people to explore the internet. Such a situation may foster conspiracy theories, such as ones about the coronavirus being created by someone in order to limit freedom and manipulate people [32]. Many governments take action and try to be informative by setting up official websites or delegating such tasks to other official institutions (such as the Robert Koch Institute in Germany). Our results show that information on coronavirus is most frequently posted on information (News) and governmental (dominated by USA and Australia) sites. Almost all of the information was true. The words “COVID-19” and “Coronavirus” were more common on governmental and news websites, while “SARS-CoV-2” was more typical for scientific journals and professional websites.

The results obtained in our study correspond to the results of the team of Okan O. et al. conducted in Germany in the period from 30 March 2020 to 7 April 2020 in the form of an online survey, where issues related to COVID-19 were discussed [33]. The study concerned the respondents’ assessment of the ease of access to reliable content on COVID-19. Researchers have confirmed the importance of local authorities in preventing the spread of the infopandemic [33].

### 4.2. Characterization and Analysis of Online Information Returned by Google Based on the “COVID-19”, “Coronavirus” and “SARS-CoV-2” Keywords

Information related to the “COVID-19”, “Coronavirus”, “SARS-CoV-2” keyword searches referred to the following topics: preventive measures against infection, epidemiological data, symptoms of the diseases/virus, infection risk factors, transmission routes. A large share (48%, 256/532) of the information that was classified as other and included issues like regulations during the pandemic, services or the economy, referred also to the etiology of SARS-CoV-2. There were notable differences between the frequency of information on websites describing transmission routes: from 5% (3/60) in Australia to 52% (31/60) in Poland. Importantly, countries with the highest number of infections (Italy and France) had a low share of websites about symptoms. In Singapore, most content on COVID-19 referred to epidemiological data while Australia, Italy and France had the highest share of sites with preventive measures. This may be credited to actions taken by governments as a result of the growing number of infections. These results for Italy are in line with the work of Rovetta and Bhagavathula (2020) who found that the 5 most important searches related to health in Italy were: face masks, amuchina (disinfectant gel), new virus symptoms, health bulletin and vaccines against coronavirus [34].

Most of the countries covered by this analysis reported their first cases of COVID-19 during the last week of January 2020. Poland was the last to report its first case, on 4 March 2020. The disease spread at a different pace in each country, which was related to various factors such as the regional structure of the demography, density of the population, social mobility or government policy. The number of infected people around the world increased threefold between 1 and 27 April 2020 from 940.5 thousand to over 3 million. The largest growth from 220 thousand to over 1 million was reported in the United States. On the 1 April, at least 100 thousand infections were identified in three of the analyzed countries: United States, Italy and Spain. In three weeks, this list has expanded to six countries, adding France, Germany and UK. The pandemic had the lowest pace in Australia, which on 1 April reported an approximately 30% increase from 5048 to 6720 infections. Meanwhile, the number of COVID-19 related deaths around the world surged from 48.5 thousand to 211.5 thousand, with the United States leading the statistics (6394 to 56,796). At the end of April 2020, more than 20,000 deaths were reported by Italy, Spain, France and UK. The COVID-19 mortality rate was 6.9% on 27 April 2020 with the highest rates in France (14.3%), Italy (13.5%) and UK (13.4%), and the lowest in Singapore (0.1%) and Australia (1.2%) [13]. It is notable that there were significant differences in this result between countries when it comes to the typology of websites and the information they contained. This analysis showed that Australia and Poland had the lowest infection rates per 1 m citizens (264 and 311, respectively) along with the largest share of content about prevention. This suggest that the effectiveness of preventive measures in both countries was relatively high.

### 4.3. Characterization and Analysis of the Online Information Returned by Google Based on the “Fake News” Keywords

An important fact is that almost 90% (137/153) of information about fake news in the COVID-19 pandemic emphasized that it referred to fake news. Three news reports (1 in Australia and 2 in Italy) could not be reliably classified. Most commonly, SERPs for the “fake news” keyword described examples of fake news on the Internet and social media. Countries with the highest number of infections (Italy, Germany, USA, France) also had the highest share of fake news about the coronavirus pandemic. In a similar period (31 December 2019–30 April 2020), a study on fake news on the Internet was conducted in Italy. This study used the BuzzSumo program and examined social media: Facebook, Pinterest, Reddit and Twitter. As with our study, researchers manually rated each post for its truthfulness [16].

The WHO has published 19 explanations and clarifications to the most frequent fake news on COVID-19 and created a simple infographic [35]. The French media reported in February 2020 that the government organized a meeting of major social media actors attended by representatives of Google, Qwant, Facebook, Twitter, Instagram, TikTok and LinkedIn and numerous government officials. The objective was to facilitate cooperation between government and online media [36]. The strategy implemented by the French seems to be reflected in these results as most of the information referred to prevention or regulations during the pandemic. The 20 SERPs for the “fake news” keyword included websites that described and corrected 152 fake news pieces.

### 4.4. Frequency of Fake News on Webpages from the “COVID-19”, “Coronavirus”, “SARS-CoV-2” Keyword Searches

It should be noted that the results indicate a low prevalence of fake news about the coronavirus pandemic in the Google search engine during the analyzed period. This is in contrast to the results of Heidi Oi-Yee Li et al., who found that more than a quarter of the most viewed YouTube videos on COVID-19 contained misleading information, reaching millions of viewers worldwide [37]. This raises a question of whether Google search provides more accurate information and minimizes the spread of misinformation. It would suggest its significant role in managing the COVID-19 pandemic. Huynh Dagher S et al. used similar tools in their study to determine the influence of the media coverage and government policies on Google searches of the symptoms of COVID-19 in the six countries: the United States, the UK, France, Italy, Spain and Germany. The keywords selected for this purpose required translation into the respective national languages, which could then have influenced the results. Dagher et al. concluded that that Internet users were influenced by the media coverage and governmental policies [38]. Zeng K et al. covered six countries: the United States, Spain, Italy, Taiwan, South Korea and Singapore, and concluded that early digital intervention had a strong correlation with the successful containment of COVID-19 [39].

There were already some initiatives aimed at fighting fake news even before the pandemic. The European Commission set up a high-level group of experts (“the HLEG”) in 2018 that started working on a report about disinformation and the principles of forwarding information [40]. The UK published its own report on fake news [41]. Another British initiative referred to creating the International Grand Committee on Disinformation and “Fake News” that involved representatives from Argentina, Belgium, Brazil, Canada, France, Ireland, Latvia and Singapore. As a result, the “Principles of the Law Governing the Internet” declaration was signed in November 2018 [42]. Singapore implemented in June 2019 the “Protection from online falsehoods and manipulation” act, making it illegal to publish false information and implementing penalties. The fight against fake news was intensified after the outburst of the pandemic [43] and these results seem to reflect that. A number of institutions and social media companies such as Facebook or Twitter implemented measures during the pandemic aimed at preventing fake news from spreading. Examples include adding automatically WHO links to every post about the coronavirus. WhatsApp decided to limit forwarding of messages in order to combat fake news [6]. Considering the results of the Abd-Alrazaq et al., 2020 [14], which identified major topics on COVID-19 published by Twitter users, such actions and measures are crucial when it comes to combating spread of false information.

### 4.5. Analysis of Associations between Results and Epidemiological Data on COVID-19

There were over 927,000 recoveries from COVID-19 around the world up to 27 April 2020 and 211,500 deaths which accounted for 81.2% of closed cases [11,13]. The highest rate was reported in Singapore (98.7%), Australia (98.5%) and Germany (94.9%) [11,13]. However, as countries employed different policies of infections control and reported various number of tests performed, the real data may be different. The highest number of tests per 1 m was performed in Italy (over 29,000) with the lowest in France (7.100) [11,13]. The study results suggest that the higher the number of SARS-CoV-2 infections is in a country, the lower is the amount of information on fake news referring to prevention. On the other hand, the higher was the number of tests in a country, the larger was the amount of information on fake news related to prevention. The higher frequency of articles indicating false information online is a positive phenomenon as it indicates effectiveness of actions taken by policy makers against fake news and disinformation.

### 4.6. Practical Implications and Future Research

The intention of this study was to use the methodology described by Arif et al. [7] with regards to information on COVID-19. The results suggest that investigating information from the Google search engine (SERP) may be a useful tool for public health information screening in cyberspace. Along with an increasing number of infections, there is a growing amount of information on prevention, treatment and consequences of a disease published by governmental, health, science or media organizations. This is also true for unreliable content circulated to the public [6,7,14,32,34,44]. The authors modeled the methodology used in the study to investigate vaccinations [7]. The research team found that it may also be used for other purposes in analyzing online information such as the global COVID-19 pandemic. This study provides a broader view on the issue of information available online about the pandemic and covers 4 continents, which makes it more representative.

Most of the countries took measures aimed at fake news on COVID-19 [24,45]. However, not all of them were equally effective. Tapia investigated fake news on COVID-19 in the Dominican Republic and indicated that the political crisis and turmoil caused by “fake news” hampers the effective response of the authorities [46]. It is also necessary that major media take responsibility for providing proper and correct content. Journalists must be aware of their important role as false or inaccurate information may have serious consequences. Last but not least, health professionals should cooperate with mass media to counter harmful myths. Effective cooperation and communication between all involved parties seems crucial in eliminating fake news [47,48]. Thus, it is necessary to continue and expand measures and actions taken at a national and global level aimed at fighting disinformation. The public health should play a crucial role in this area.

### 4.7. Limitations

First of all, this is a qualitative study that lacks representativeness and thus should not be generalized [27]. Nevertheless, its wide scope may be considered a strength. It covered countries from 4 continents: Europe, North America, Asia and Australia. Six out of the 9 analyzed countries had the highest number of infections as of 27 April 2020. This research referred to crucial elements of the healthcare system and public health such as social exposure to health risks from coronavirus fake news. The content analysis during the pandemic was very wide and allowed us to thoroughly investigate the issue of fake news. Another limitation is cultural differences between the societies of individual countries, which were not taken into account. The diversity of topics related to the pandemic in different countries and the way they are communicated shows the importance of differences in the perception of reality. Such differences among inhabitants of different regions despite the use of the same language were already indicated in a study by Hughes et al. in 2021 [49].

The SERPs were classified according to 7 different topics, judged true or false, and each website was evaluated according to 17 elements. The SERPs for the “fake news” keyword were checked as to whether the fake news was corrected and explained. The study was conducted in a short period of time that covered the pandemic peak and time in which governments implemented various measures. The team analyzed 685 SERPs. Although Arif N. et al., 2018, investigated the first 100 websites for each country [7], this analysis covered an entirely new issue for the world; thus, the original assumptions had to be changed. The lower number of SERPs than originally planned may stem from government policies aimed at fighting disinformation. UK had 61 SERPs, Spain 76, United States 74 and Poland 73. However, researchers believe that the 685 SERPs still make it a reliable result. A similar study using SERPs was also carried out in March 2020. Here, too, a review of the first 30 SERPs was assumed, using previous research results [50] on Internet usage, showing that 90% of users are limited to the first 30 addresses [51]. The purpose of this study was to evaluate online patient educational material on COVID-19 for understandability. Hernández-García and Giménez-Júlvez analyzed 80 weblinks in total on COVID-19 for the United States and Spain [44].

The important limitation is the location from which the study was conducted. The computer’s location may affect the results; however, the research team implemented measures in order to mitigate a risk of biased results. The authors did not use a VPN during research, which made the results more neutral. It is also essential there were not any adds-on in the browser. Another limitation of this study is that we only looked at webpages and did not investigate social networks. In addition, we used the same, neutral, keywords without taking into account potential differences in the most searched terms used in national languages. It is likely that users could find more biased information by using more negative or positive search terms. The study may also be subject to information bias that may have occurred during data collection (content analysis). Due to the nature of the study, the fundamental error that could have occurred was a misclassification bias and observer/interviewer bias. However, it is acceptable that most of research is subject to some degree of misclassification [52].

The information on celebrities in relation to COVID-19 may be subjective as it frequently referred to health policy decision makers; thus, it was up to Google algorithms to decide whether information was related to a celebrity or something else, such as, for example, the economy. It did not, however, influence other results.

The researchers also attempted to find associations between the frequency of new articles about the coronavirus on different types of websites and epidemiological data for the countries; nevertheless, the low number of analyzed countries in terms of statistical requirements made it impossible to find statistical significance.

When undertaking the study, the researchers assumed that there would be more fake news in the first months of the pandemic than there actually were. Due to the fact that the study was conducted in the initial period of the pandemic, it would be worth repeating it using the same methodology. Currently, fake news related to COVID-19 has been the subject of number of publications. Their main purpose was usually identifying and controlling fake news [53,54,55,56].

## 5. Conclusions

The analyzed content about COVID-19 returned by Google during the pandemic showed that governments in the analyzed countries took effective measures in fighting fake news during the pandemic. Most of the published information was available on news or governments sites, referred to prevention, epidemiological data or disease symptoms. There were differences between continents when it came to the types of information available online: Asia was dominated by epidemiological data, Western Europe and Australia by prevention, and North America and Central Europe by risk factors. The COVID-19 pandemic information, including fake news, rarely made reference to celebrities, religion or testimonials. The JAMA score was comparable in most of the analyzed countries, except for Singapore and USA, where it was higher.

Although the first 20 SERPs from the COVID-19, coronavirus and SARS-CoV-2 keywords contained true information, it is inevitable that false information did make its way to the Internet when analyzed more deeply. Most commonly, SERPs for the “fake news” keyword described examples of fake news on the Internet and social media. In countries with the highest number of tests, a higher frequency of information on fake news referring to prevention was found. The higher the number of SARS-CoV-2 infections, the lower the amount of information on fake news referring to prevention against infection with the virus.

## Figures and Tables

**Table 1:** Search engine results page (SERP) according to web pages typology (*n* = 685).

Country	“Key Word”	Government (G)	Health Portal (HP)	News (N)	Non-Profit (NP)	Professional (P)	Commercial (C)	Scientific Journal (SJ)	TOTAL SERP
Spain	COVID-19	7	0	5	2	4	2	0	20
	Coronavirus	3	1	14	1	0	1	0	20
	SARS-CoV-2	3	3	4	2	6	1	1	20
	Fake news	0	0	10	0	1	5	0	16
	total	13	4	33	5	11	9	1	76
Singapore	COVID-19	5	0	4	2	1	9	0	21
	Coronavirus	2	0	12	2	1	2	1	20
	SARS-CoV-2	2	1	0	0	0	6	11	20
	Fake news	0	0	14	3	0	3	0	20
	total	9	1	30	7	2	20	12	81
USA	COVID-19	11	0	2	2	1	4	0	20
	Coronavirus	13	0	1	1	4	1	0	20
	SARS-CoV-2	2	1	0	0	2	4	11	20
	Fake news	0	0	9	1	0	4	0	14
	total	26	1	12	4	7	13	11	74
Australia	COVID-19	15	0	4	0	1	0	0	20
	Coronavirus	12	0	5	1	1	0	1	20
	SARS-CoV-2	4	0	5	2	5	3	1	20
	Fake news	0	0	18	0	2	0	0	20
	total	31	0	32	3	9	3	2	80
Poland	COVID-19	4	3	10	0	0	3	0	20
	Coronavirus	2	2	13	0	1	2	0	20
	SARS-CoV-2	4	3	9	0	3	1	0	20
	Fake news	0	0	5	1	1	6	0	13
	total	10	8	37	1	5	12	0	73
UK	COVID-19	2	0	2	7	4	4	0	19
	Coronavirus	2	0	14	0	2	1	1	20
	SARS-CoV-2	1	2	0	0	1	3	5	12
	Fake news	0	0	8	1	0	1	0	10
	total	5	2	24	8	7	9	6	61
Germany	COVID-19	5	1	10	0	2	2	0	20
	Coronavirus	0	0	18	0	2	0	0	20
	SARS-CoV-2	5	0	4	0	11	0	0	20
	Fake news	1	0	19	0	0	0	0	20
	total	11	1	51	0	15	2	0	80
Italy	COVID-19	6	0	5	0	5	3	0	19
	Coronavirus	6	0	12	0	1	1	0	20
	SARS-CoV-2	2	0	11	0	7	0	1	21
	Fake news	1	0	18	1	0	0	0	20
	total	15	0	46	1	13	4	1	80
France	COVID-19	4	0	14	0	1	1	0	20
	Coronavirus	3	0	16	0	1	0	0	20
	SARS-CoV-2	4	0	9	2	4	0	1	20
	Fake news	0	0	20	0	0	0	0	20
	total	11	0	59	2	6	1	1	80
TOTAL N		131	17	324	31	75	73	34	685
TOTAL %		19%	2%	47%	5%	11%	11%	5%	100%

Values indicate the number of webpages in each SERP.

**Table 2:** Composition of the search engine result page (SERP) by typology of webpages for keywords: “COVID-19”, “Coronavirus”, “SARS-CoV-2”.

	G	HP	N	NP	P	C	SJ	TOTAL
**Spain**	13 (22%)	4 (7%)	23 (38%)	5 (8%)	10 (17%)	4 (7%)	1 (2%)	60 (100%)
**Singapore**	9 (15%)	1 (2%)	16 (26%)	4 (7%)	2 (3%)	17 (28%)	12 (20%)	61 (100%)
**USA**	26 (43%)	1 (2%)	3 (5%)	3 (5%)	7 (12%)	9 (15%)	11 (18%)	60 (100%)
**Australia**	31 (52%)	0 (0%)	14 (23%)	3 (5%)	7 (12%)	3 (5%)	2 (3%)	60 (100%)
**Poland**	10 (17%)	8 (13%)	32 (53%)	0 (0%)	4 (7%)	6 (10%)	0 (0%)	60 (100%)
**UK**	5 (10%)	2 (4%)	16 (31%)	7 (14%)	7 (14%)	8 (16%)	6 (12%)	51 (100%)
**Germany**	10 (17%)	1 (2%)	32 (53%)	0 (0%)	15 (25%)	2 (3%)	0 (0%)	60 (100%)
**Italy**	14 (23%)	0 (0%)	28 (40%)	0 (0%)	13 (22%)	4 (7%)	1 (2%)	60 (100%)
**France**	11 (18%)	0 (0%)	39 (65%)	2 (3%)	6 (10%)	1 (2%)	1 (2%)	60 (100%)

Color intensity indicates percentage values-the darker the color, the higher the percentage: up to 20%, >30%, >40% and >50%. G—Government, H—Health Portal, N—News, NP—Non-Profit, P—Professional, C—Commercial, SJ—Scientific journal.

**Table 3:** Frequency of different types of fake news according to countries (N, %).

Fake News Topic	Spain	Singapore	USA	Australia	Poland	UK	Germany	Italy	France	Total	
Coronavirus developed by humans (e.g., in Wuhan, in the Pasteir Instutute in France, by Americans)	3	3	5	5	3	1	7	3	11	41	9%
“Miraculous” ways of prevention (e.g., Drinking alcohol or hot water every 15 min, heroin intake, drinking bleach, vitamin C, holding breath)	6	13	12	33	8	11	18	26	18	145	32%
Spread of the virus (e.g., Home animals, mosquitos, wrappings, distance between people, deliveries from China)	-	2	-	12	5	1	2	12	5	39	9%
Underestimating the virus (Coronavirus does not exist; just another flu)	-	1	1	2	2	-	8	2	-	16	4%
“Harmful” technologies that favor the pandemic (5G)	-	1	2	8	3	2	5	6	3	30	7%
Children, youth and black people are resistant to COVID-19 (disease attacks seniors only)	-	1	-	3	1	1	1	2	1	10	2%
“Effective” treatment (e.g., antibiotics treatment)	1	7	8	3	9	-	8	8	1	45	10%
Local sensations referring to the virus (e.g., swans in Venice, elephants in tea plantations)	8	5	5	3	7	3	14	7	4	56	13%
Instructions on how to avoid the virus (e.g., Troops blocking cities, bodies in street, disinfection from air, quarantining politicians on an island)	1	2	1	-	-	-	-	-	-	4	1%
Other (BCG vaccine protects against coronavirus, flu vaccines and other)	3	1	14	6	7	3	11	8	9	62	14%
Total	22	36	48	75	45	22	74	74	52	448	100%

**Table 4:** Examples of debunked fake news.

Country	Example
Poland	“Coronavirus is fake”: commercial encouraging to ignore quarantine. “Please, be informed that according to the special act on coronavirus, your funds will be transferred to the Polish Central Bank. Log in in order to keep your 1000 PLN”.
Singapore	Millions of Facebook users continue to be exposed to coronavirus misinformation: some of the most dangerous falsehoods had received hundreds of thousands of views, including claims like “black people are resistant to coronavirus” and “Coronavirus is destroyed by chlorine dioxide”. A message circulating on messaging platforms and social media claiming that an Enterprise Singapore safe-distancing ambassador had fined someone for sitting on a seat that was marked out as part of safe-distancing measures is false.
UK	Journalists and so-called experts have seriously suggested that the SARS-CoV-2 coronavirus at the heart of the epidemic could have been produced in the Level 4 Biosafety Laboratory (BL4) in China’s Wuhan. The 5G conspiracy theory—which alleges, among other things, that COVID-19 has either been caused by the frequencies used for the new wireless technology, or that those signals impair the human immune system. “(…) don’t send your loved ones to hospital because if you do it, chances are they will not return home alive”.
USA	(…) the coronavirus is a plot to hurt President Trump—A theory pushed by some at Fox News heavily at first. The Chinese Foreign Ministry spokesperson’s retweet of an article blaming the U.S. for infecting Wuhan with coronavirus went viral, viewed 160 million times within hours; but where did the story come from? Viral posts wrongly suggest that the COVID-19 death toll is exaggerated because “the state” has instructed that “anyone who didn’t die by a gunshot wound or car accident” be listed as a coronavirus victim. Experts say there is no such default classification—and that the U.S. death count is probably underestimated.
Spain	el virus había sido fabricado en realidad por un laboratorio canadiense, y después robado por dos espías chinos (the virus was developed in Canada and then stolen by Chinese spies). el agua caliente elimina el virus y que tomar agua cada 15 minutos ayuda a eliminar los virus que hayan entrado por la boca (hot water eliminates the virus; so if you drink it every 15 min, you will kill it).
Germany	Das Virus töte nicht allein, sondern nur im Verbund mit anderen Krankheiten (The virus kills only in the case that there are other accompanying diseases). Der Rauch von Feuerwerk würde gegen das Virus helfen (Fireworks smoke will help fight the virus).
France	Se raser la barbe pour soigner le coronavirus (Shaving a beard protects against coronavirus). Le nouveau coronavirus créé en 2004 par l’institut Pasteur (The new virus was developed in 2004 by the Pasteur Institute). Des “petites bombes” pour tuer le virus vont être lâchées à 3 heures du matin par avion (“’Small bombs’ to kill the virus will be dropped at 3 am by plane”).
Italy	l’italia si trasformi a terra conquista tramite il fenomeno “5G” che va contenuto e controllato controllato (Italy is transforming itself into a land conquered through the technology “5G” that must be contained and controlled). “La crisi italiana porta a tutti i cittadini buoni regalo alimentari per un valore di 200 euro” (The Italian crisis brings food gift vouchers worth 200 euros to all citizens).
Australia	Chinese people are not converting to Islam because of the outbreak. (…) use of hand sanitizers contributed to cancer. The latest coronavirus has been found in energy drinks.

Some of the content was very long; therefore, the omitted text was marked as: “(…)”.

**Table 5:** Examples of whether a testimonial, celebrity, religion was mentioned.

SERPs for the “COVID-19”, “Coronavirus”, “SARS-CoV-2” Keywords		
Country	Type	Example
Poland	testimonial	“Jason Hargrove, a bus driver from Detroit, was irritated with passengers coughing all the time and posted a video in which he called for hygiene during the pandemic. He died 11 days later due to the coronavirus”.
Singapore	NA	
UK	testimonial	“A museum boss says he had hallucinations filled with “visions of snakes” after being struck down by coronavirus”.
USA	celebrity	“In London, British Prime Minister Boris Johnson has been moved into intensive care. The announcement came a day after he was admitted to a London hospital with what his office called “persistent symptoms of coronavirus””.
Spain	religion	“La peregrinación a La Meca —hach— es el viaje que deben emprender los fieles musulmanes al menos una vez en la vida. (…) Y de riesgo, sobre todo en tiempos del COVID-19. No hay sector inmune al coronavirus; el comercio, el deporte, la cultura e incluso la fe son víctimas del patógeno, que ya ha causado la cancelación del Carnaval de Venecia, las Fallas de Valencia y decenas de giras”.
Germany	testimonial	“Über seine persönliche Situation sagt der 40-Jährige im Interview mit spot on news: “Für mich ist es nicht so schlimm. Ich muss beim Einkaufen nicht zwangsläufig kommunizieren und sehe ja noch die Augen und die Augenbrauen. Das hilft. Bei anderen wichtigen Sachen, wie zum Beispiel beim Arzt, kann ich es aufschreiben”. Und was hält er von Mundschutzmasken mit Sichtfeld? “Im Krankenhaus würde ich mir wünschen, dass alle transparente Masken tragen. So kann man die Menschen lachen sehen-es ist wichtig, die Emotionen weiterhin zu sehen””.
France	testimonial	“« On a arrêté de travailler pour respecter la discipline du confinement, alors qu’on doit manger et nourrir nos enfants, explique le père de famille âgé de 30 ans. Pour moi, c’était être indiscipliné ou partir. » Alors avant l’aube, il a pris ses deux enfants et un vélo chargé de maigres bagages pour rejoindre la ville d’Antsirabe, sa femme et le reste de sa famille, à plus de 150 kilomètres et trois jours de marche de là”.
	celebrity	“« Ça n’a jamais été dans mon tempérament de pousser des coups de gueule, j’ai plutôt tendance à prendre le temps de discuter, d’expliquer les choses avec pédagogie. Mais, cette fois, je me suis rendu compte qu’on avait un poids trop faible, j’étais obligé de réagir. » C’est sur les réseaux sociaux, qu’ Arnaud Assoumani, multimédaillé paralympique au saut en longueur et au triple saut, a finalement manifesté son ras-le-bol après le report des Jeux olympiques de Tokyo en raison de l’épidémie de coronavirus”.
	celebrity	“Un exercice facile à réaliser et, qui selon Sharon Stone, peut être utile pour se protéger des maladies”. “Cela permet à la cage thoracique de s’étirer à leur capacité maximale pour vous rendre plus forts. Alors je veux que vous fassiez ça pour renforcer vos poumons, vos côtes pour être prêt à affronter ce qu’il faudrait”, a-t-elle déclaré”.
Italy	testimonial	“Per lo Stato sono classificati come ‘microimpresa’, ma per i bambini sono una seconda casa. Eppure questi luoghi colorati, pieni di disegni appesi alle pareti, dove migliaia di piccoli hanno mosso i primi passi, e grazie ai quali altrettante mamme hanno potuto dedicarsi al proprio lavoro, rischiano di non riaprire quando l’emergenza Covid19 sarà rientrata”.
	testimonial	“«Ma 102 anni sono pochi, posso prestargliene qualcuno, anzi no meglio che ognuno si tenga suoi» scherza al telefono. «Ne ho vissute tante, sì. Ho visto la guerra e i tedeschi fare del male, c’era la paura». E il coronavirus? «Ne ho viste così tante che di questo coronavirus quasi non me ne sono accorta. Prima facevo la postina a Bergamo, poi qui andavo a fregare i pavimenti, facevo le pulizie nelle case. Ho sempre lavorato”.
	celebrity	“Nel Regno Unito intanto il premier Boris Johnson—risultato positivo—è stato ricoverato e il Times scrive che in ospedale ha ricevuto ossigeno. Ieri sera la Regina Elisabetta ha parlato alla nazione: un evento raro, capitato solo 4 volte in 68 anni di regno”.
Australia	celebrity	“’The Prime Minister has been moved this evening from intensive care back to the ward, where he will receive close monitoring during the early phase of his recovery’, a Downing Street spokesman said today”.
SERP for the “Fake News” Key Word		
Country	Type	Example
Poland	NA	
Singapore	testimonial	“Coronavirus fake news: Kenyan woman ‘killed off’ by false WhatsApp rumour. WhatsApp message (…) give information about the death of Uganda’s fourth coronavirus victim, but the picture used in the post was of Elsie, a Kenyan woman living in London”.
	celebrity	“Cristiano Ronaldo and the pope tested positive”.
UK	celebrity	“Fans who believed Ronaldo to be sick might still have been thrilled to shake his hand; and no one is besieging virology labs to demand the truth”.
USA	NA	
Spain	NA	
Germany	religion	“Da es vermehrt zu Fake-News zu diesem Thema kam, noch einmal deutlich: Der Veranstalter hat der Stadt #Dortmund schon vor einiger Zeit mitgeteilt, dass die Veranstaltung Festi Ramazan 2020 in den @Westfalenhallen nicht stattfindet”.
France	celebrity	““L’intégralité des cours manqués à cause du COVID-19 seront donc rattrapés pendant les vacances d’été”. La nouvelle est apparue sous la forme d’une capture d’écran d’un prétendu tweet d’Emmanuel Macron. Il s’agit évidemment d’un faux, puisque le compte à l’origine de la publication est @EmmanuelMecron”
Italy	NA	
Australia	religion	“Chinese people are not converting to Islam because of the outbreak”.

Some of the content was very long; therefore, the omitted text was marked as: “(…)”. NA—not applicable.

## Data Availability

Data is contained within the article or Supplementary Materials.

## Supplementary Materials

The following supporting information can be downloaded at: https://www.mdpi.com/article/10.3390/ijerph19031491/s1, Table S1: Types of information from Google using the “COVID-19”, “Coronavirus” and “SARS-CoV-2” keywords, by country (*n* = 532); Table S2: Types of information about COVID-19 returned by Google using the “fake news” keyword, by country (*n* = 153).

## Abbreviations

C	Commercial
CDC	Centers for Disease Control and Prevention
CFR	Case Fatality Ratio
G	Government
GIS	Chief Sanitary Inspectorate “Główny Inspektorat Sanitarny”
HP	Health Portal
MZ	Ministry of Health “Ministerstwo Zdrowia”
N	News
NHS	National Health Service
NP	Non-Profit
SERP	search engine result page
SJ	Scientific journal
P	Professional
WHO	World Health Organization
