# Supplementary material for: Online Information of COVID-19: Visibility and Characterization of Highest Positioned Websites by Google between March and April 2020—A Cross-Country Analysis
Source: Int J Environ Res Public Health. 2022 Jan 28;19(3):1491. doi: 10.3390/ijerph19031491 (PMC8835343; doi:10.3390/ijerph19031491)
Supplement: Supplementary file 1 [file ijerph-19-01491-s001.zip › Table S1.pdf]

Table S1. Types of information from Google using the “COVID-19”, “Coronavirus” and “SARS-CoV-2” keywords, by country (n = 532)

Country	"Keyword"	Type of information															Was the information true or false according to scientific knowledge?		TOTAL SERP
		Quarantine	Symptoms of disease /infection	Disease or infection risk factors	Disease or infection consequences	Ways of virus transmission	Incubation period	Carrying virus	Treatment	Preventive measures	Epidemiological data (number of infections, deaths etc.)	Contained a testimonial (e.g., a personal story)	Mentioned celebrity	Mentioned religion	Other: regulations, services, economy etc.	About online fake news	True	False	
Spain	COVID-19	3	3	3	3	4	3	2	2	2	6	0	0	1	16	1	20	0	20
	Coronavirus	7	6	7	4	5	2	3	4	7	12	0	4	0	14	1	20	0	20
	SARS-CoV-2	3	12	15	11	15	11	8	11	11	7	0	0	0	13	0	20	0	20
	total	13	21	25	18	24	16	13	17	20	25	0	4	1	43	2	60	0	60
Singapore	COVID-19	5	1	5	1	0	0	0	1	4	8	0	0	0	15	1	21	0	21
	Coronavirus	2	2	2	2	2	1	1	4	3	11	0	2	0	12	1	20	0	20
	SARS-CoV-2	1	3	5	3	5	9	9	2	6	7	0	0	0	11	1	20	0	20
	total	8	6	12	6	7	10	10	7	13	26	0	2	0	38	3	61	0	61
USA	COVID-19	7	11	13	9	11	6	4	5	11	11	0	1	0	17	0	19	0	20[a]
	Coronavirus	5	8	9	3	6	3	1	1	11	7	0	0	0	17	1	20	0	20
	SARS-CoV-2	1	7	5	2	9	8	6	3	1	7	0	0	0	14	1	20	0	20
	total	13	26	27	14	26	17	11	9	23	25	0	1	0	48	2	59	0	60
Australia	COVID-19	0	6	3	0	0	0	1	1	8	7	0	0	0	15	0	20	0	20
	Coronavirus	1	5	6	1	0	0	0	1	10	7	3	1	0	14	0	20	0	20
	SARS-CoV-2	0	3	2	1	3	1	2	0	7	3	0	0	0	11	0	20	0	20

	total	1	14	11	2	3	1	3	2	25	17	3	1	0	40	0	60	0	60
Poland	COVID-19	1	9	8	6	9	0	5	2	6	9	1	0	0	9	0	20	0	20
	Coronavirus	9	6	4	5	14	2	4	1	16	9	1	0	0	9	0	20	0	20
	SARS-CoV-2	2	12	3	1	8	3	3	1	8	5	0	0	0	2	2	20	0	20
	total	12	27	15	12	31	5	12	4	30	23	2	0	0	20	2	60	0	60
UK	COVID-19	5	7	6	2	7	3	2	8	6	7	0	0	1	5	0	19	0	19
	Coronavirus	4	8	7	2	3	4	1	0	8	7	2	0	0	10	0	20	0	20
	SARS-CoV-2	3	6	6	0	3	0	1	1	2	3	0	0	0	1	0	12	0	12
	total	12	21	19	4	13	7	4	9	16	17	2	0	0	16	0	51	0	51
Germany	COVID-19	0	13	7	7	8	1	0	2	8	7	2	0	0	11	0	20	0	20
	Coronavirus	15	11	14	0	4	1	7	0	7	11	2	0	0	14	0	20	0	20
	SARS-CoV-2	2	8	7	1	3	2	1	1	7	7	0	0	0	15	0	20	0	20
	total	17	32	28	8	15	4	8	3	22	25	4	0	0	40	0	60	0	60
Italy	COVID-19	0	1	1	1	1	1	0	2	8	5	0	1	0	11	0	20	0	20
	Coronavirus	0	3	1	1	3	1	1	2	10	10	3	2	0	7	0	20	0	20
	SARS-CoV-2	0	8	4	2	7	3	6	8	7	2	0	0	0	7	5	20	0	20
	total	0	12	6	4	11	5	7	12	25	17	3	3	0	25	5	60	0	60
France	COVID-19	0	3	3	0	2	0	0	2	5	7	6	1	0	9	0	20	0	20
	Coronavirus	1	4	5	0	3	2	0	6	8	7	2	2	0	11	0	20	0	20
	SARS-CoV-2	1	5	5	1	6	4	2	3	9	3	0	0	0	6	0	20	0	20
	total	2	12	13	1	11	6	2	11	22	17	8	3	0	26	0	60	0	60
TOTAL N		61	139	128	61	126	67	62	71	174	167	18	14	1	256	14	531	0	532
TOTAL %		11%	26%	24%	11%	24%	13%	12%	13%	33%	31%	3%	3%	0%	48%	3%	100%	0%	100%

[a] Webpage contained suspicious information indicating a fraudulent attempt, quote "(...) The Recording Academy® and its affiliated charitable foundation MusiCares® have established a COVID-19 Relief Fund to help our peers in the music community affected by the Coronavirus pandemic."; source: https://www.grammy.com/musicares/get-help/musicares-coronavirus-relief-fund

[b] Values indicate the number of webpages in each SERP.
