# Supplementary material for: Online Information of COVID-19: Visibility and Characterization of Highest Positioned Websites by Google between March and April 2020—A Cross-Country Analysis
Source: Int J Environ Res Public Health. 2022 Jan 28;19(3):1491. doi: 10.3390/ijerph19031491 (PMC8835343; doi:10.3390/ijerph19031491)
Supplement: Supplementary file 1 [file ijerph-19-01491-s001.zip › Table S2.pdf]

Table S2. Types of information about COVID-19 returned by Google using the "fake news" keyword, by country (n = 153)

Country	Keyword	Fake news on: Quarantine	Symptoms of disease /infection	Disease or infection risk factors	Disease or infection consequences	Ways of virus transmission	Incubation period	Carrying virus	Treatment	Preventive measures	Alternative/complementary medicine (unconventional methods of treatment)	Stance toward complementary and alternative medicine (CAM): positive	neutral	negative	Epidemiological data (number of infections, deaths etc.)	Whether the page contained a testimonial (e.g., a personal story)	Whether a celebrity was mentioned	Whether religion was mentioned	Other, ex. regulations during pandemic, services, economy	Information about online fake news	Was fake news explained: Yes	No	Not applicable	TOTAL SERP
Spain	Fake news	0	0	0	0	0	1	0	0	1	0	0	0	0	0	0	0	0	11	16	12	0	3	16 [d]
Singapore	Fake news	0	1	3	1	5	0	0	5	5	1	3	0	0	5	2	2	0	16	20	14	0	6	20 [e]
USA	Fake news	1	1	3	2	3	0	2	1	1	2	1	0	0	3	0	1	0	12	14	14	0	0	14 [f]
Australia	Fake news	0	0	5	2	1	0	4	1	9	0	0	0	0	1	0	2	1	2	18	19	1[a]	0	20
Poland	Fake news	1	0	0	0	3	0	1	1	6	2	0	0	2	1	0	0	0	1	12	10	0	3	13 [g]
UK	Fake news	0	0	2	0	3	0	0	3	1	2	1	0	0	0	0	1	0	0	10	10	0	0	10
Germany	Fake news	0	0	7	1	0	0	0	5	10	2	0	0	0	1	0	2	2	7	20	20	0	0	20
Italy	Fake news	0	0	3	5	3	0	0	8	11	0	0	0	0	0	0	0	0	5	20	18	2[b,c]	0	20
France	Fake news	0	0	0	0	2	1	1	1	9	0	0	0	0	0	0	5	0	2	20	20	0	0	20
TOTAL N		2	2	23	11	20	2	8	25	53	9	5	0	2	11	2	13	3	56	150	137	3	12	153
TOTAL %		1%	1%	15%	7%	13%	1%	5%	16%	35%	6%	3%	0%	1%	7%	1%	8%	2%	37%	98%	90%	2%	8%	100%

a. The article denied information about a government official who reportedly went to a hospital with a suspected infection. Rather, he was quarantined on an island. https://www.thebigsmoke.com.au/author/fake-news/

b. The article presented a new WhatsApp policy, https://www.tgcom24.mediaset.it/tgtech/fake-news-sul-coronavirus-whatsapp-limita-le-catene_17028242-202002a.shtml

c. The article about readers' attitudes, scientific activities, statements of politicians https://www.tgcom24.mediaset.it/tgtech/fake-news-sul-coronavirus-whatsapp-limita-le-catene_17028242-202002a.shtml

d. Other SERPs did not refer to the pandemic. Out of 16 SERPs, three were neutral, ex. "Coronavirus: How can you stop the spread of misinformation?". In one case the authors had doubts whether the fake news was explained: https://www.elplural.com/politica/espana/vox-abona-bulos-denuncia-fake-news-medios_236979102

e. Out of 20 SERPs, six were neutral, ex. "A collection of 400 scientists have joined together to form Indian Scientists' Response to COVID-19 – committed to debunking false information about the virus.", "Facebook, Twitter, and YouTube have each moved quickly to remove coronavirus misinformation that encourages people to take actions that could put them at risk (...). "

f. Other SERPs did not refer to the pandemic. Last 3 SERPs referred to Africa, India and Ecuador

g. Out of 13 SERPs, three were neutral ex. "how to read online information and detect fake news"

h. Values indicate the number of webpages in each SERP mentioning a topic.
